# Un purpura thrombopénique amégacaryocytaire acquis qui cache une leucémie aigue myéloblastique

**DOI:** 10.11604/pamj.2017.26.32.9215

**Published:** 2017-01-23

**Authors:** Hicham Eddou, Ali Zinebi, Abdelaziz Khalloufi, Mohammed Sina, Mehdi Mahtat, Kamal Doghmi, Mohammed Mikdame, Mohammed Karim Moudden, Mohammed El Baaj

**Affiliations:** 1 Service de Médecine Interne, Hôpital Militaire Moulay Ismail, Meknès, Maroc; 2 Faculté de Médecine et de Pharmacie de Fès, Maroc; 3 Service d’Hématologie Biologique, Hôpital Militaire Moulay Ismail, Meknès, Maroc; 4 Service d’Anatomopathologie, Hôpital Militaire Moulay Ismail, Meknès, Maroc; 5 Service d’Hématologie Clinique, Hôpital Militaire d’instruction Mohammed V, Rabat, Maroc

**Keywords:** Amégacaryocytose acquise, purpura thrombopénique, leucémie aigue, Acquired amegakaryocytosis, thrombocytopenic purpura, acute leukemia

## Abstract

Le purpura thrombopénique amégacaryocytaire acquis est une pathologie très rare caractérisé par une thrombopénie sévère liée une réduction ou une disparition des mégacaryocytes au niveau de la moelle osseuse. Il peut être primaire idiopathique ou secondaire à de nombreux états pathologique dont des hémopathies. Nous rapportons le cas d'un patient de 24 ans admis pour prise en charge d'un syndrome hémorragique mis sur le compte d'un purpura thrombopénique immunologique. Le diagnostic a été redressé en une amégacaryocytose aquise après un échec de la corticothérapie et la réalisation d'un myélogramme. Le patient a été mis sous traitement par ciclosporine avec une évolution rapide vers une leucémie aigue myéloblastique. La progression d'une amégacaryocytose acquise vers une leucémie aigue est rapporté mais généralement pas aussi rapidement et surtout précéder par un syndrome myélodysplasique ou une aplasie médullaire. Cette observation impose un suivi strict et rapproché de ces pathologies d'apparence bénigne.

## Abstract (en)

Acquired amegakaryocytic thrombocytopenic purpura is a very rare condition characterized by severe thrombocytopenia linked to the reduction or disappearance of megakaryocytes in the bone marrow. It may be primary idiopathic or secondary to many pathological conditions including hematologic disorders. We report the case of a 24-year-old patient admitted for haemorrhagic syndrome caused by immunological thrombocytopenic purpura. The diagnosis was acquired amegakaryocytosis after the failure of corticotherapy and the performance of myelography. The patient was treated with ciclosporin with rapid progression to acute myeloblastic leukemia. The progression of acquired amegakaryocytosis to acute leukemia is reported but it is generally not so rapid and above all it is preceded by myelodysplastic syndrome or medullary aplasia. This study highlights the importance of a close follow-up of these pathologies with a benign-like appearance.

## Introduction

Le purpura thrombopénique amégacaryocytaire acquis (PTAA) ou amégacaryocytose acquise, initialement décrit par Korn [1], est une entité très rare caractérisé par une thrombopénie sévère liée une réduction ou une disparition des mégacaryocytes au niveau de la moelle osseuse alors que les autres lignées sont normales [2]. Cette affection peut être primaire ou secondaire à de nombreux états pathologiques [3]. Nous rapportant dans ce travail l'observation d'un patient présentant une PTAA rapidement évoluant vers une leucémie aigue.

## Patient et observation

**Figure 1:**
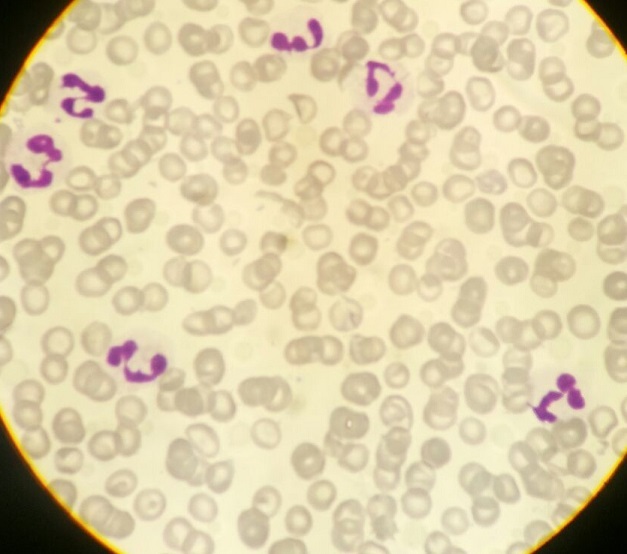
Frottis sanguin confirmant la présence d’une thrombopénie (coloration MGG (x100))

**Figure 2:**
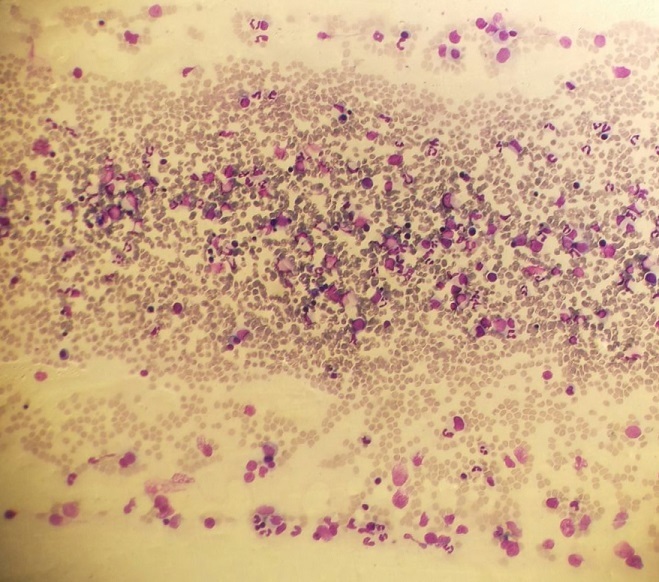
Frottis médullaire montrant une cellularité normal avec absence de mégacaryocytes (coloration MGG (X 40))

**Figure 3:**
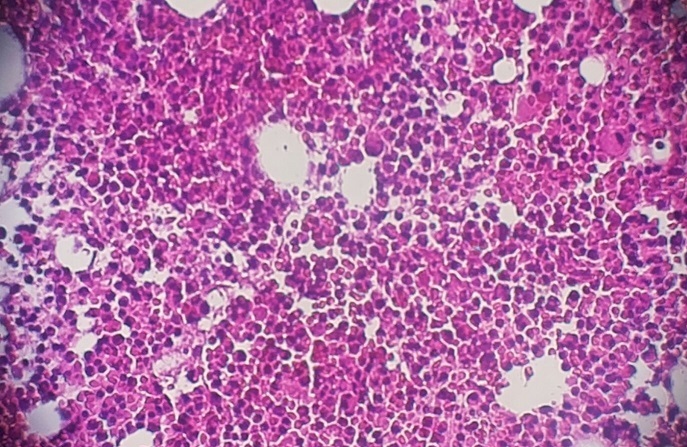
Coupe d’une biopsie ostéo-médullaire montrant une moelle de richesse normal avec absence de mégacaryocytes (HE x 100)

**Figure 4:**
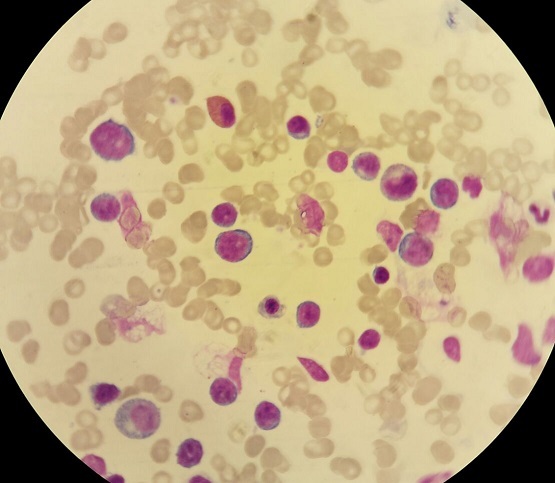
Frottis médullaire montant un envahissement par des blastes granulaires (coloration MGG (x 100))

Il s'agit d'un patient de 24 ans, sans antécédents pathologiques notables, qui est admis pour prise en charge d'une épistaxis récidivante et des lésions cutanées purpuriques évoluant depuis une quinzaine de jours avec conservation de l'état général. L'examen clinique confirme la présence d'un syndrome hémorragique cutanéo-muqueux isolé. Il n'y avait pas de syndrome anémique, tumoral ou infectieux associé. L'hémogramme note une thrombopénie profonde et isolée à 9 Giga/l avec des taux d'hémoglobine et de globules blancs normaux. Le frottis sanguin confirme la réduction du taux des plaquettes (Figure 1) sans anomalie morphologique des autres lignées. Le bilan d'hémostase, le bilan hépatique, le dosage des vitamines B9 et B12, les sérologies virales (Hépatite (B, C) et HIV) ainsi que le bilan immunologique (anticorps anti-nucléaire, anti DNA natif et anti-Sm) sont revenu normaux. Le diagnostic d'un purpura thrombopénique immunologique a été retenu et le patient est mis sous traitement corticoïde (bolus de méthyl-prednisolone 15mg/kg/j pendant 3 jours puis relais par prednisone à la dose de 2 mg/kg/j) mais sans réponse. Cette cortico-résistance a motivé la réalisation d'un myélogramme qui a montré une moelle de richesse normal avec réduction extrême des mégacaryocytes alors que les lignées érythroblastique et granuleuses sont quantitativement et qualitativement normales (Figure 2). Une biopsie ostéo-médullaire avec étude histochimique a confirmé la diminution de la lignée mégacaryocytaire sans infiltration tumoral (Figure 3). Le scanner thoraco-abdomino-pelvien était normal. Le diagnostic a été redressé en purpura thrombopénique amégacaryocytaire acquis pour le quel le patient est mis sous traitement immunosuppresseur à base de ciclosporine à la dose de 5mg/kg/j avec un début de réponse dès la 6ème semaine (taux de plaquette à 50 giga/l). A 3 mois du début de traitement, le patient présente un tableau infectieux fait d'une fièvre et des frissons avec un point d'appel pulmonaire. La radiographie pulmonaire révèle un foyer basal droit. L'hémogramme montre une pancytopénie (anémie normochrome normocytaire arégénérative à 90 g/l, thrombopénie à 20 Giga/l et une neutropénie à 0,9 Giga/l sans hyperleucocytose associée). Un frottis sanguin et médullaire montre une infiltration par 30% de blastes de taille petite à moyen qui ont un rapport nucléo-cytoplsmique élevé et dont le cytoplasme est basophile renfermant quelques granulations et parfois un ou deux corps d'Auer évoquant une transformation en une leucémie aigue myéloblastique (Figure 4). La cytométrie au flux confirme le diagnostic en montrant l'infiltration par des blastes exprimant le Myéloperoxydase en intracytoplasmique, le CD117, le CD33 et le CD13. Le caryotype médullaire était normal. Le patient a bénéficié d'une chimiothérapie d'induction à base de Daunorubicine et d'Aracytine compliqué d'une septicémie à acinotobacter Baumani résistant à une large antibiothérapie. Le décès est survenu à J18 du début de la chimiothérapie dans un tableau de choc septique.

## Discussion

**Point du vue du biologiste:** Le PTAA est une maladie très rare caractérisée par une thrombopénie isolée qui est souvent sévère (numération plaquettaire <20-30 × 10^9^/L), suite à une diminution marquée ou à l´absence totale de mégacaryocytes dans la moelle osseuse. En en raison de sa rareté et de sa nature hétérogène, les mécanismes pathogéniques de cette maladie ne sont pas bien définis et son étiologie est susceptible d´être variée. L'étude de survie des plaquettes marquées par le chrome chez des patients atteints de PTAA ne montrent aucun signe de destruction prématurée ou de séquestration. Les études de culture cellulaire in vitro ont identifié, par contre, un défaut intrinsèque des cellules progénitrices de la lignée mégacaryocytaire (CFU-MK). D'autre études suggère une inhibition ou une destruction immunitaire des mégacaryocytes que sa soit par voie humorale ou cellulaire [4]. Le PTAA peut être primaire idiopathique ou survenir en association avec des troubles lymphoprolifératifs, des maladies auto-immunes (surtout un Lupus érythémateux disséminée), des infections, des tumeurs solides, des carence en vitamine B12 ou des excès de consommation de drogues et d'alcool. Le PTAA peut également être la première manifestation de troubles de la moelle osseuse précédant un syndrome myélodysplasique ou une aplasie médullaire [4]. Une progression d'un PTAA vers une leucémie aigue est rapporté mais généralement pas aussi rapidement, comme le cas de notre observation, et surtout précéder par un syndrome myélodysplasique ou une aplasie médullaire. Dans ces derniers cas, le PTAA peut s'associer à la présence d'anomalies cytogénétiques clonales acquises, une macrocytose ou des signes de dysérythropoïèse et sa différenciation avec un syndrome myélodysplasique unilignée peut s'avérer très difficile [5, 6].

**Point de vue du clinicien:** Sa prévalence est inconnue et la littérature reste limitée à des rapports de cas. On note une légère prédominance féminine et bien qu'il peut toucher n'importe quel âge, la plupart des cas rapportés survient chez des sujets d'âge moyens ou âgés [7]. Sur le plan clinique, le PTAA se présente habituellement par un syndrome hémorragique cutanéo-muqueux. La splénomégalie est généralement absente. L'hémogramme révèle généralement une thrombopénie isolée mais peut s'associer à une anémie en cas de saignement important. A la différence du PTI, qui constitue le principal diagnostic différentiel, le myélogramme trouve une diminution importante ou une absence des mégacaryocytes. En raison de la diversité des mécanismes physiopathologiques, un large éventail de thérapies est utilisé chez les patients avec PTAA avec des degrés de réponses variables. De plus la rareté de cette pathologie ne permet pas pour le moment une conduite thérapeutique standardisée. La première étape du traitement consiste à corriger un facteur de risque réversible, tel une infection ou un excès de consommation d´alcool. Le recours à un support transfusionnel plaquettaire peut être indiqué dans certaines situations hémorragiques à risques notamment cérébrales. Contrairement au PTI, et comme c'est le cas de notre patient, l'utilisation d'une corticothérapie en monothérapie reste généralement inefficace pour traiter un PTAA (bien que certains groupes ont fait état de succès avec cette approche) [8]. Les autres conduites font appel à des immunosuppresseurs (cyclosporine A, cyclophosphamide, vincristine, immunoglobuline polyvalente, serum anti-lymphocytaire et le rituximab), la splénectomie ou une greffe de moelle osseuse [9]. Mais là aussi la preuve d'efficacité reste faible et se base essentiellement sur des rapports de cas. L´utilisation des analogues de thrombopoietine (Romiplostin ou Eltrombopac) dans les PTAA réfractaire semble pour le moment un choix logique [10].

## Conclusion

Le PTAA reste une pathologie rare aux mécanismes physiopathologiques intriqués et dont la conduite thérapeutique standard reste à définir. L'évolution rapide, en moins de 4 mois, de notre patient vers une leucémie aigue et sans phase de myélodysplasie nous impose un suivi strict et régulier de ces pathologies d'apparence bénigne.

## References

[1] Korn D (1962). Congenital hypoplastic thrombocytopenia. Am J Clin Pathol..

[2] Gewirtz AM, Hoffman R (1990). Human megakaryocyte production: cell biology and clinical considerations. Hematol Oncol Clin North Am..

[3] Trimble MS, Glynn MFX, Brain MC (1991). Amegakaryocytic thrombocytopenia of 4 years duration: successful treatment with antithymocyte globulin. Am J Hem..

[4] Lown R, Rhodes E, Bosworth J, Shannon MS, Stasi R (2010). Acquired amegakaryocytic thrombocytopenia: potential role of thrombopoietin receptor agonists. Clin Adv Hematol Oncol..

[5] Felderbauer P, Ritter PR, Mattern D, Schmitz F, Bulut K, Ansorge N (2004). Acquired pure megakaryocytic aplasia: a separate haematological disease entity or a syndrome with multiple causes. Eur J Haematol..

[6] Lai DW, Loughran TP, Maciejewski JP, Sasu S, Song SX, Epling-Burnette PK (2008). Acquired amegakaryocytic thrombocytopenia and pure red cell aplasia associated with an occult large granular lymphocyte leukemia. Leuk Res..

[7] Erkurt MA, Kaya E, Baran M, Tmen EY, Fienel S, Kuku R (2005). Rapid progression of acquired amegakaryocytic thrombocytopenia to myelodysplastic syndrome: case report. Turk J Haematol..

[8] Mulroy E, Gleeson S, Chiruka S, Danazol (2015). Effective option in acquired Amegakaryocytic thrombocytopaenic purpura. Case Rep. Hematol..

[9] Mirzania M, Khalili S, Hasanpoor A, Shamshiri RA (2014). Anti-CD20 antibody is effective in the patient with refractory amegakaryocytic thrombocytopenia, 25 months follow up. Int J Hematol Oncol Stem Cell Res..

[10] Cela I, Miller IJ, Katz RS, Rizman A, Shammo JM (2010). Successful treatment of amegakaryocytic thrombocytopenia with eltrombopag in a patient with Systemic Lupus Erythematosus (SLE). Clin Adv Hematol Oncol..

